# How to form and close the brain: insight into the mechanism of cranial neural tube closure in mammals

**DOI:** 10.1007/s00018-012-1227-7

**Published:** 2012-12-15

**Authors:** Yoshifumi Yamaguchi, Masayuki Miura

**Affiliations:** Department of Genetics, Graduate School of Pharmaceutical Sciences, The University of Tokyo, and CREST, JST, 7-3-1 Hongo, Bunkyo-ku, Tokyo, Japan

**Keywords:** Neural tube closure, Brain development, Tissue fusion, Exencephaly, Live imaging

## Abstract

The development of the embryonic brain critically depends on successfully completing cranial neural tube closure (NTC). Failure to properly close the neural tube results in significant and potentially lethal neural tube defects (NTDs). We believe these malformations are caused by disruptions in normal developmental programs such as those involved in neural plate morphogenesis and patterning, tissue fusion, and coordinated cell behaviors. Cranial NTDs include anencephaly and craniorachischisis, both lethal human birth defects. Newly emerging methods for molecular and cellular analysis offer a deeper understanding of not only the developmental NTC program itself but also mechanical and kinetic aspects of closure that may contribute to cranial NTDs. Clarifying the underlying mechanisms involved in NTC and how they relate to the onset of specific NTDs in various experimental models may help us develop novel intervention strategies to prevent NTDs.

## Introduction

The process of cranial neural tube closure (NTC) creates the basic morphological scaffold for the central nervous system. Defects in this critical process result in lethal cranial neural tube defects (NTDs), most commonly expressed in humans as anencephaly. In human, NTDs including spinal defects such as spina bifida and craniorachischisis, occur in approximately one out of 1,000 births worldwide. NTDs have been studied intensively both epidemiologically in humans, and experimentally in animal models, including frogs, chickens, and rodents [1–4]. Although dietary folic acid fortification or supplementation efforts have been effective in preventing NTDs in human populations, little is known about how it works [5–7]. Understanding the basis of normal and abnormal NTC is not only fascinating from a biological perspective but also has important clinical relevance.

There are many excellent reviews on NTC mechanisms in mammals (see [2–4, 8, 9]). Herein, we summarize previous and recent studies addressing the molecular and cellular mechanisms of cranial NTC in amniotes such as birds and mammals. It is now obvious that many signaling pathways and morphogenetic processes are evolutionary conserved among birds and mammals, although many differences exist as well. As it is more feasible to perform experimental manipulation in order to dissect molecular and cellular pathways in chicken than in mouse, studies on the morphogenetic mechanisms using chicken as well as those using mice greatly help to increase our understanding of mammalian cranial NTC. We will also discuss the questions and concepts that could be useful in further understanding various NTD mutant phenotypes and in developing approaches for future studies of cranial NTC.

## Mechanisms of cranial NTC

### Tissue movement in mammalian cranial NTC

**Fig. 1:**
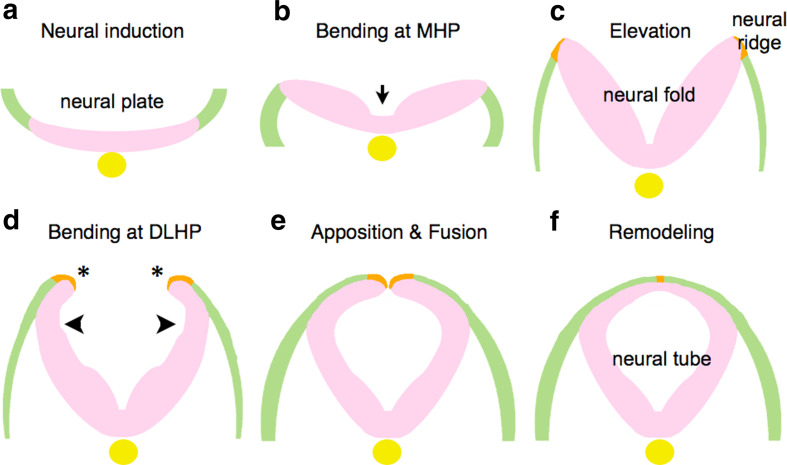
Morphological changes of neural plates to neural tube. After neural induction (**a**), the neural plate bends at MHP (**b**) and is elevated to form the neural fold (**c**). Subsequently, flipping of the edges (*asterisks*) and bending at DLHP occur (**d**), resulting in apposition and fusion of the edges (**e**). Remodeling takes place to separate neuroectoderm and surface ectoderm (**f**). Neuroectoderm (neuroepithelium): *pink*. Non-neural ectoderm (surface ectoderm): *green*. Boundary region within non-neural ectoderm: *Orange*. Notochord: *yellow*

Cranial NTC in mammals as well as in other vertebrates begins after neural induction that discriminates the neural plate from the adjacent surface ectoderm (Fig. 1a), and is achieved through sequential changes in the morphology of the neural plate as follows [2, 10].

1. *Elevation and bending:* The neural plate changes its morphology by bending in two phases, each initiated at “hinge points” [2]. The plate begins as a largely horizontal, although slightly convex, dorsal neuroectodermal field (Fig. 1b). The first morphological change is the bending of the plate at the midline, which forms the medial hinge point (MHP) (Fig. 1b, arrow), thus dividing the neural plate into bilaterally symmetric regions. These lateral regions are then elevated by intrinsic neuroectodermal cell movements, and possibly by the extrinsic expansion of the underlying cranial mesenchyme as well, to create the vertical, concave walls of neuroepithelium that make up the neural folds (Fig. 1c). At the same time, the neural plate elongates rostrocaudally through convergent extension and cell division (see a later section; “Neurulation and body axis elongation through convergent extension, the PCP pathway, and oriented cell division”).
  In the second phase, the neural folds bend at paired dorsolateral hingepoints (DLHP) (Fig. 1d). The exact location varies along the rostral-caudal axis.
2. *Apposition and fusion:* Once the neural folds are elevated and have bent at both the MHP and DLHP, the tips of the neural folds are flipped (Fig. 1d, asterisks) and can be apposed (Fig. 1e). In apposition, the neural folds meet at the dorsal midline, after which the epithelium fuses by “zipping” or “buttoning-up” to form the neural tube.
3. *Remodeling:* Once the tube is closed, the dorsal midline is remodeled to separate the inner neuroectoderm, or neuroepithelium, from the outer non-neural ectoderm (the surface ectoderm or future epidermis) (Fig. 1f).

**Fig. 2:**
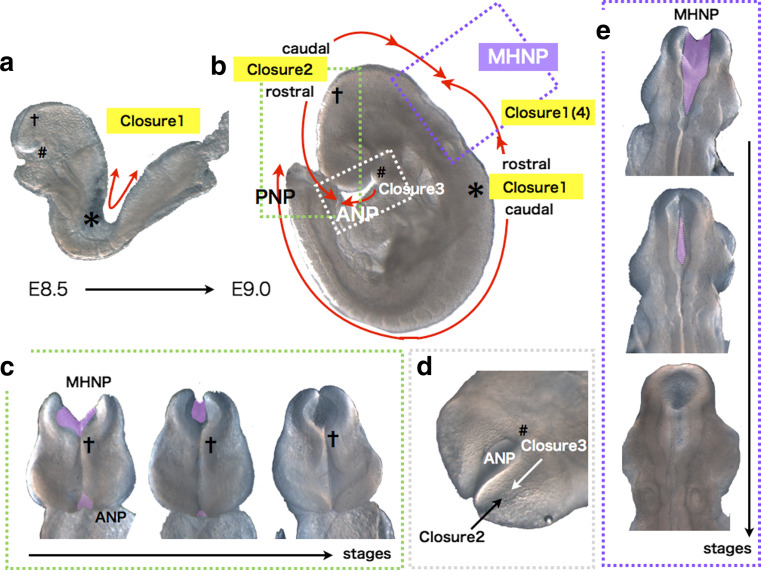
Multiple closures in cranial NTC of mouse embryos. **a** Bi-directional closure 1 occurs from the cervical region (*asterisks*) before embryonic turning in an E8.5 ICR embryo. **b** Schematic representation of multiple closures in an E8.75 ICR embryo. MHNP is closed by caudal closure 2 and rostral closure 1(closure 4), and ANP by caudal closure 2 and closure 3. *asterisks*: closure 1 start site. *Crosses*: bi-directional closure 2 start site. *Sharp*: closure 3 start site. Directions of the closures are shown by *red arrows*. **c** Frontal views of closure 2 at MHNP and ANP. **d** Ventral view of closure 3 at ANP. **e** Dorsal views of closure 1(4) and 2 at MHNP. Unclosed regions are colored *purple*

The above basic processes are observed commonly among vertebrates except fish, which forms neural keels before neural tube formation, but the mode and mechanism of cranial NTC appears most complicated in amniotes, especially in mammals [11]. In most mouse strains, the above process of closure is initiated at several points along the neural tube, at different developmental stages [8, 12–17]. At E8.5, when embryos have 6–7 pairs of somites (somite stage [ss] 6–7), the tips of the neural ridges typically have met and fused at the midline of the hindbrain/cervical boundary (Fig. 2a, b, shown by asterisk (*)), and neural tube fusion proceeds both rostrally (toward the hindbrain) and caudally (toward the trunk) (summarized in [10, 13]). Closure initiated from this point is termed closure 1 (Fig. 2a, b). Around ss 10–13, the neural ridges meet and fuse at the forebrain/midbrain boundary (FMB), initiating closure 2, which also proceeds bi-directionally from the contact point (Fig. 2a–c, shown by cross (†)). A third fusion initiation point begins at the rostral end of the neural plate. This closure 3 proceeds caudally and meets the rostrally directed wave of closure 2 to seal the anterior neuropore (ANP) (Fig. 2a, b, d, shown by sharp (#)). The caudally directed closure 2 meets the rostrally directed closure 1 (which is also sometimes referred to in the literature as closure 4 [14–16]) to seal the midbrain-hindbrain neuropore (MHNP) (Fig. 2b, e). Analogous multiple closure sites are also observed in other mammals including humans, and also in a bird [8, 18–20].

Any disturbance in the dynamic, sequential events of cranial NTC can cause cranial NTDs [10]. In particular, failure in closure 2 often causes exencephaly. The exencephalic brain often grows well in utero, but eventually undergoes neurodegeneration and the ultimate anencephalic phenotype ensues [8, 21, 22]. Defective closure 1 between the midbrain and lower spine causes craniorachischisis, in which the neural tube is open along the entire axis of the body secondary to a complete failure of the neural folds to elevate and fuse. A partial failure in closure 1 to close the thoracic or lumbosacral region, or its re-opening, causes the common human birth defect spina bifida.

### Morphogens affecting the position of bending

**Fig. 3:**
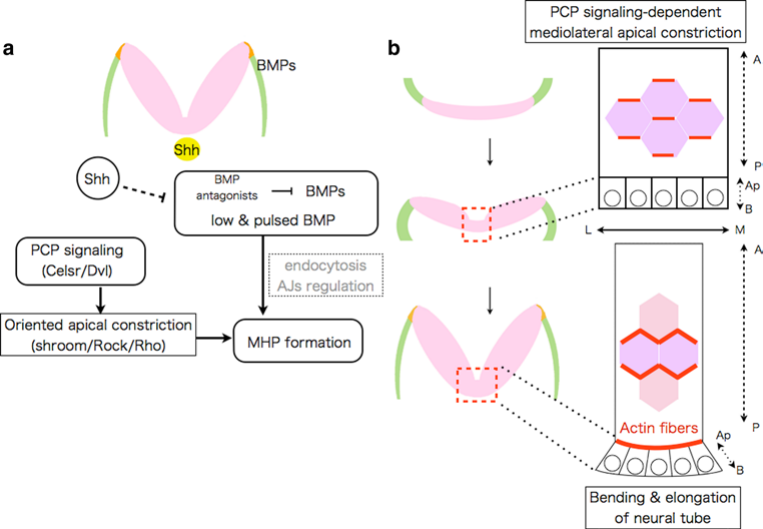
Mechanisms of bending at MHP in the cranial regions identified in chicken embryos. **a** Signals involved in MHP formation, and mechanisms of their actions in chicken cranial region. **b** PCP signaling links convergent extension with neural plate bending via oriented apical constriction in chicken cranial region. Oriented apical constriction along mediolateral (*M*–*L*) axis within neuroepithelial cells (actin fibers are shown with *red*) couples elongation of the neural plate along anteroposterior (*A*–*P*) axis with its bending along *M*–*L* axis

Neural fold bending at the MHP and DLHP are essential steps in cranial NTC. However, the precise mechanism(s) by which the bending position is determined at the molecular and cellular levels has remained unclear. A recent study using chicken embryos revealed how the MHP is determined [23]. Within neural plates, a two-dimensional canonical BMP activity gradient exists, which results in a low and pulsed BMP activity at the MHP. Disturbing this gradient by overexpression BMP signaling antagonists (e.g., Noggin) can induce ectopic hinge-point formation in the more lateral neural plates, and conversely, overexpression of a constitutively active form of BMP receptor IA suppresses MHP formation. Thus, BMP blockage is necessary and sufficient for MHP formation in the chicken cranial region. Because BMP blockage does not affect the expression of *Shh,* or *phoxA2*, one of the ventral neural plate markers, the study suggests that the effect of BMP blockage on MHP formation is likely independent of Shh. How the BMP activity gradient is formed still remains unclear. The study also proposed that BMP attenuation induces neural plate bending via apical constriction, possibly through endocytosis of apical protein Par3 and N-cadherin [23, 24] (Fig. 3a). Further studies are required to examine whether similar BMP gradient is important for mammalian NTC. Interestingly, some of BMP signaling mutants including *Noggin* exhibit NTD in mice [25–27].

Compared to the MHP, it seems more difficult to understand how the position of DLHP formation is determined in the cranial regions. This is because position of the DLHP shifts during NTC and differs among species, and there are no known DLHP-specific molecular markers at present. The elevation and bending mechanisms of DLHP have been well studied in the mouse spinal region, where the structure is relatively simple and therefore, more feasible for analysis. Precise observations of relationships between gene expression patterns and DLHP formation in the neural folds suggested that integrative actions between Shh, BMPs, and the BMP antagonist Noggin, regulate the formation of the DLHP [27, 28]. Spina bifida and exencephaly are seen in mice overexpressing *Shh* or lacking *Noggin*, suggesting that a similar regulatory interaction likely operates in the cranial region, where bending of the DLHP is also a prominent event during neurulation [25–27]. As well as in the MHP, the ultimate mechanism by which these signals actually causes the neural plates to bend, remains to be revealed.

#### Do growth factors regulate neural plate bending directly or through neural plate patterning?

**Fig. 4:**
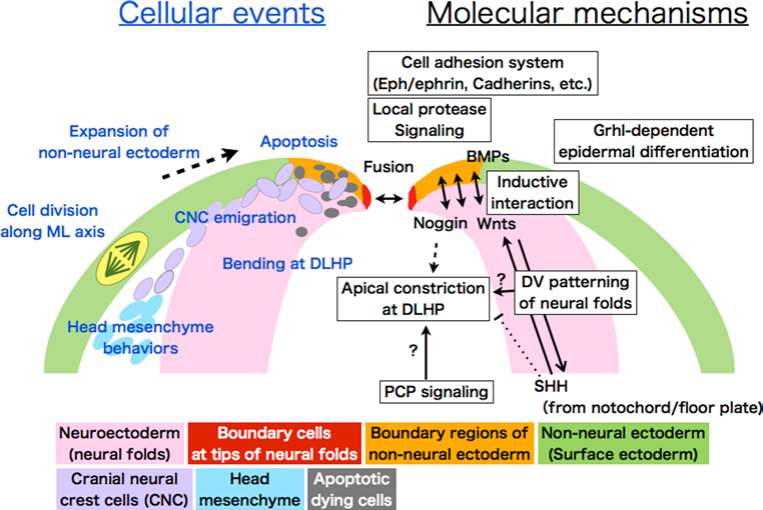
Schematic illustration of events occurring in the dorsal neural folds during cranial NTC. Cellular behaviors and molecular mechanisms are shown with *blue* and *black fonts*, respectively. Neuroectoderm (neuroepithelium): *pink*. Non-neural ectoderm (surface ectoderm): *green*. Boundary region within non-neural ectoderm: *orange*. Boundary cells mediating fusion at tips: *red*. Cranial neural crest cells (CNC): *purple*. Head mesenchyme: *light blue*. Apoptotic dying cells: *gray*. A cell undergoing division is shown with *yellow* in surface ectoderm (*left*)

With regard to the position of bending, in addition to morphogenetic movements, we should also consider dorsoventral (D–V) patterning of the neural folds. It is well known that the above-mentioned growth factors including Shh are crucial for D–V patterning of the neural tube. The nature of neural progenitors in the neural tube is specified gradually during development by a combination of transcriptional factors’ expression, which is generated by morphogen gradients; Shh as the ventralizing factor, and Wnts and possibly BMPs as the dorsalizing factors [29, 30] (Fig. 4). The D–V patterning starts at the neural plate stage when the MHP begins to form, since Shh emanating from the notochord is already inducing a ventral identity (Nkx6.1-positive cells) in the medial region of the neural plate, and is acting continuously as a morphogen to progressively pattern the neural folds, at least in mouse [29, 31, 32].

In the mouse cranial region, if the dorsal neural folds are ventralized by either overactive Shh signaling or the suppression of dorsalizing signals such as Wnt, the consequences are often a deformed neural tube lacking DLHP formation, which results in cranial NTDs [26, 32–36]. Likewise, mice deficient for transcriptional factors that are expressed in dorsal neural folds, including *paired box 3* (*Pax3*), *Zic2*, *Zic5*, and *sal*-*like 2* (*Sall2*), also exhibit cranial NTDs [27, 37–40]. However, it appears that even if the neural folds are dorsalized by loss of Shh signaling or enhanced Wnt signaling, the DLHP forms and NTC is completed [28, 35, 41]. These lines of evidence suggest that specification of dorsal neural folds is crucial for success in cranial NTC (Fig. 4). This raises several questions: What downstream factors characterize the hinge point formation site? Is there a specific border that marks DLHP formation along the dorsoventral (or, mediolateral) axis? In other words, is there a combination of transcription factors or a crosstalk of signaling activities that determines where hingepoints form? Answering these questions will require the precise characterization of the position of hingepoint formation along the D–V and rostrocaudal axis in the cranial neural folds.

### Neurulation and body axis elongation through convergent extension, the PCP pathway, and oriented cell division

The cellular movement of convergent extension occurs during gastrulation and neurulation in vertebrates [42]. Convergent extension leads to the rostrocaudal extension of the body axis, driven by polarized cell rearrangement, including lateral-to-medial cell displacement within the tissue and cell intercalation at the midline. Convergent extension is governed by evolutionarily conserved planer cell polarity (PCP) signaling, which was originally identified to regulate cell polarity within the plane of the wing epithelium in *Drosophila*. Defective PCP signaling causes NTDs in vertebrates [43–45]. A severe NTD, craniorachischisis, is found in several mouse PCP-signaling mutants, including *loop*-*tail* (*Lp*) [*Vangl2*] mutants, *crash* [*Celsr1*] mutants, *circletail* [*Scribble1*]*, dishevelled 1* (*Dvl1*) and *Dvl2* double mutants, and *frizzled 3* (*Fz3*) and *Fz6* double mutants [45–50]. In these PCP-signaling mutants, the neural plate and the underlying notochord fail to elongate rostrocaudally, due to ineffective cell intercalation at the midline. As a result, the neural plate widens, hampering the apposition and contact of the neural folds at the midline, and eventually leading to NTDs [43, 50].

In addition to convergent extension, there would be another factor contributing to body axis elongation; cell division. In *Lp* mutants, defective elongation in the midline is seen mainly in the caudal notochordal region and to a lesser extent in the rostral region [51]. This suggests that rostrocaudal elongation in the most anterior neural plate is relatively independent of convergent extension, and may instead result primarily from extensive longitudinally oriented mitoses occurring in the midline [51–54]. Whereas amphibian and fish do not significantly increase the embryonic cell numbers during NTC, higher vertebrates such as birds and mammals do substantially increase the cellular population of the neural tissues [52, 55]. Thus, inclusion of cell division into potential mechanisms involved in cranial NTC is plausible in birds and mammals.

### Mechanics of neural plate bending: links between the PCP pathway and apical constriction

Among the cellular mechanisms that bend the neuroepithelial sheet, the contraction of subapical actin microfilaments in neuroepithelial cells is the most-studied intrinsic NTC mechanism [56–59]. Actin microfilaments (F-actin) accumulate to form a meshwork at the apical cortex which then contract, reducing the apex of the neuroepithelial cells during NTC. The contraction is driven by the molecular motor myosin. Several studies have shown that disrupting actomyosin with chemicals causes cranial NTDs [58, 60–62]. Similarly, mice deficient for regulators of the cytoskeleton present with exencephaly (*Abl1/Abl2*, *n*-*Cofilin*, *Marcks*, *Mena*, *Mlp*, *Shroom*, *Palladin*, and *Vinculin*) [63–73]. In contrast, NTC in the spinal region does not appear to require actomyosin [58, 60].

During NTC, the neural plate bends only along the mediolateral axis. Such a polarized neural plate bending implies a polarized cellular contraction, otherwise the neural plate bending would occur only radially. A recent study using chicken embryos revealed that PCP signaling directly links apical constriction to convergent extension, promoting the polarized mediolateral bending of the neural plate (Fig. 3b) [74]. During the bending process Celsr1, a vertebrate homolog of the *Drosophila* gene *Flamingo* (one of the core PCP members) [49], concentrates in adherens junctions (AJs) oriented along the mediolateral axis of the neural plate, along with Dvl, DAAM1, and PDZRhoGEF, which together upregulate Rho kinase. This causes actomyosin-dependent planar-polarized contraction, which promotes the simultaneous apical constriction and midline convergence of the neuroepithelial cells (Fig. 3b). This system ensures that neural plate bending and body axis elongation are well coordinated [74, 75]. A similar mechanism may also operate in mammalian NTC.

Besides these intrinsic neural plate mechanisms, NTC is also affected by extrinsic factors from surrounding tissues, the earliest of which is Shh emanating from the notochord. Signals from the head mesenchyme and non-neural surface ectoderm also shape morphogenetic events during cranial NTC.

### Head mesenchyme’s role in closure

The cranial neural plate is surrounded mainly by the head mesenchyme, which originates from primary mesenchyme, the earliest group of cells ingressing upon gastrulation [76]. The head mesenchyme possibly affects cranial NTC, because mutant embryos lacking genes expressed in the head mesenchyme, e.g., *twist homolog 1* (*Twist*), *cartilage homeo protein 1* (*Cart1*), *aristaless*-*like homeobox 3* (*Alx3*), or the ubiquitously expressed *HECT domain containing 1* (*Hectd1*), often have exencephaly. It is thought that this is due to defective DLHP formation and neural fold elevation of the forebrain and midbrain, along with abnormal head mesenchyme density around the neural folds [77–80]. The density of the head mesenchyme decreases in the *Twist* or *Cart1* mutant, but increases in the *Hectd1* mutant. Thus, proper head mesenchymal cell behavior is likely required to regulate cranial NTC. Understanding how head mesenchyme affects the formation of the neural folds will require future study.

### Non-neural surface ectoderm: a supporting player

An effect of the non-neural surface ectoderm on the cranial NTC was first demonstrated in urodele amphibians and birds [81, 82]. In chicken embryos, medially directed non-neural surface ectoderm expansion is observed only in the cranial region, not the spinal region, and surgically removing the tissue prevents DLHP formation. Thus, as it expands, the non-neural surface ectoderm may physically force the neural plate to bend (Fig. 4) [82–84]. Since a small, narrow boundary region of the non-neural surface ectoderm adjacent to the neural plate is both sufficient and necessary to induce bending at the DLHP in the head region in chickens, and in the lower spinal region in mice [28, 84], another possibility arises, not mutually exclusive to the first: bending at the DLHP may be mediated by inductive interactions between the neural folds and the adjacent non-neural surface ectoderm (Fig. 4). In fact, as mentioned in the previous chapter, BMPs from the non-neural surface ectoderm induce expression of the BMP antagonist *Noggin* in the tips of the neural folds, and this antagonism of BMP signaling is necessary and sufficient to form the DLHP in the lower spinal regions in mice [27].

The importance of the non-neural surface ectodermal cells for NTC is emphasized by several findings, one of which comes from a functional analysis of the *grainyhead*-*like* (*Grhl*) gene family. Grhl family genes encode transcription factors that includes the* Drosophila* gene *grainyhead* (*grh*), which is essential for epidermal differentiation and wound healing in the fly [85, 86]. Loss of the *Grhl2 or Grhl3* genes, which are specifically expressed in the non-neural surface ectoderm, causes NTDs [87–90]. Mutant of Grhl family genes interact with several of the PCP-signaling mutants, and exhibit PCP-like defects both in fly and mice [91, 92]. A series of studies indicate that the *Grhl* genes are indispensable for proper development of non-neural epithelial tissues in mice [88, 90, 92–96]. Thus, non-neural epithelial properties in the non-neural surface ectoderm defined by the *Grhl* genes are considered to be essential for successful cranial NTC (Fig. 4).

Another finding supporting the importance of non-neural surface ectoderm in cranial NTC is that mouse embryos lacking *protease*-*activated receptor 1* (*Par1*) and *Par2* develop exencephaly and spina bifida [97]. Both *Par1* and *Par2* are expressed in the non-neural surface ectoderm. *Par2* expression is restricted to the cells surrounding the neuropore, and possibly to boundary cells (Fig. 4, see below). Matriptase, a membrane-tethered protease that is activated by hepsin and prostasin activates Par2. Par2’s downstream signals include the G protein-coupled receptors Gi/z and Rac1, as shown by the finding that conditionally ablating these genes in *Grhl3*-expressing cells causes NTDs [97]. Thus, NTC requires local protease signaling in cells at the edge of the non-neural surface ectoderm (Fig. 4).

### Zipping and fusion: the non-neural ectoderm boundary plays a key role

Cells at the edge of the non-neural surface ectoderm mediate zipping and fusion to seal the midbrain and hindbrain. Classic transmission electron microscopy studies, as well as recent live-imaging studies, showed that at the border of the mesencephalon and rhombencephalon, non-neural surface ectoderm cells overlying the neural folds make the initial contact in sealing the neuropore [98–100]. These non-neural surface ectoderm cells differ from the underlying neuroectoderm and the adjacent non-neural surface ectoderm in both morphology and location; they have a bipolar shape and are aligned along the rostral-caudal axis like a chain, whereas adjacent surface ectoderm cells are polygonal [99]. These “boundary cells” are brought into proximity and then into contact by “zipping” at the rhombencephalon (rostral closure 1 and caudal closure 2) (Fig. 4) [99, 100]. Within the mesencephalon of cultured embryos, the boundary cells protrude from the epithelial layer on opposing sides of the neural folds, extend long cellular processes toward each other, and then form contacts between the juxtaposed folds [99]. This “buttoning-up” closure eventually resolves into a continuous closure.

This observation raises the possibility that the boundary cells, which are at the tips of the non-neural ectodermal cells covering the neural fold edge, are sufficient to complete fusion if they are appropriately juxtaposed prior to being zipped. To test this idea, we need an experimental innovation that allows cells from both sides to be forced into contact before normally closed while keeping the embryo and closure intact, whether in utero or in a culture system. If this approach could be achieved, it would be interesting to examine whether or not edge boundary cells can achieve fusion in various NTD mutants, such as the PCP or *Grhl2/3* mutants. This will help to determine whether NTDs arise solely from defective elevation and bending, or from the fusion process and its maintenance, as well.

### Molecules that mediate cell–cell interaction and fusion

Compared to the mechanisms for elevation and apposition, little is known about the molecules that mediate the cell-to-cell interactions responsible for neural fold fusion [2, 101]. The subtypes of classic cadherins, which are cell-adhesion molecules, differ between neuroectoderm (N-cadherin^+^) and non-neural surface ectoderm (E-cadherin^+^), and subtype switching from E-cadherin to N-cadherin in neuroepithelium occurs during NTC [102, 103]. Deletion of *N*-*cadherin* results in increased cell death in cranial neural folds during NTC [104]. Removal of *N*-*cadherin* specifically from dorsal edges of neural folds (Wnt1^+^) results in exencephaly, as well as cardiac defects caused by defects in the cardiac neural crest cells [104]. Addition of blocking antibodies to N-cadherin or antisense-oligonucleotide against E-cadherin also disrupts the cranial NTC in chicken and rat [105, 106]. These results suggest that proper regulation of these classic cadherins is indispensable for cranial NTC. Mice carrying hypomorphic alleles of p38-interacting protein (p38IP) (*drey*) exhibit exencephaly or spina bifida [107]. p38IP and p38 MAPK activation are required for downregulation of E-cadherin in mesoderm during gastrulation. It might be possible that downregulation of E-cadherin in neuroepithelium during normal NTC is also mediated by p38-dependent pathway. This regulation appears to be independent of Snail, a transcriptional factor that is a well-known regulator for switching of these cadherins in epithelial–mesenchymal transition during gastrulation [103, 108]. The mutually exclusive expression of these cadherins is likely based on a negative-feedback regulation, just as suppressing *E*-*cadherin* mRNA in cultured non-neural epithelial cell lines leads to the compensatory upregulation of N-cadherin, which is not normally expressed in those cells [109]. In *Grhl2* mutant mice, which exhibit cranial NTDs as mentioned above, *E*-*cadherin* mRNA and its protein are decreased in the epidermis, but N-cadherin protein is increased [88, 90]. Other evidence of epithelial dysregulation in *Grhl2* mutants includes decreased expression of the tight junction protein claudin-4. Since E-cadherin appears to be expressed in the non-neural ectodermal boundary cells that directly mediate zipping and fusion [90] (and our unpublished observation), a precise characterization of the behaviors and dynamics of these cells may shed light on the role of these classic adherence molecules during NTC (Fig. 4).

Another cell–cell interaction system, the Eph-ephrin system, is also important for fusion (Fig. 4). Eph tyrosine kinase receptors and their membrane-bound ephrin ligands participate in several developmental processes, including repelling axonal growth cones and promoting cell migration. The cranial neural tube fails to close in a small percentage of mice deficient in *ephrin*-*A5* or its receptor, *EphA7* [110]. *EphA7* and *ephrin*-*A5* are strongly expressed along the edge of the neural fold. *EphA7* has three splice-variant transcripts, and all of them are expressed in the neural folds. Two of the splicing variants encode a truncated form of EphA7 that lacks the tyrosine kinase domain, and these variants enhance cell adhesion with cells expressing *ephrin*-*A5* in vitro. These lines of evidence suggest that in the neural folds, the *EphA7* and *ephrin*-*A5* presumably act as a cell adhesion signal [110]. The importance of the Eph-ephrin system in fusion is also reported in spinal NTC; blocking EphA activity in whole-embryo culture delays NTC at the posterior neuropore, without disturbing neural plate elevation or DHLP formation [111].

### Boundary regions and cranial neural crest cells

Cranial neural crest cells (CNCs) are generated at the dorsal edge of the neural folds (the boundary regions) (Fig. 4). Failure of the CNCs to develop or emigrate is often observed in cases of exencephaly [3], although the mechanism how defects in CNCs lead to exencephaly is unclear. In mice, CNCs in the midbrain and hindbrain begin detaching from the edge of the neural folds and start migrating well before NTC is complete [112, 113]. A recent study reported that the non-neural surface ectoderm (*Wnt1*-*Cre* ^+^/E-cad^+^/PDGFRa^+^) in the cranial boundary regions produces CNC-like cells (Fig. 4) [114]. The “metablast” discussed in that study is likely a CNC subpopulation previously considered to arise only from neuroectoderm [114].

Because properties of the boundary regions are important for successful closure, it should be interesting to examine whether CNC-defective mutants disrupting the cranial NTC have defects also in the boundary regions. Thus, it has become evident that cranial NTDs can be caused by disrupted signaling or cellular events in the boundary regions (Fig. 4), including CNCs’ emigration, and cell death as follows.

### Programmed cell death in the boundary regions

#### Apoptosis

Programmed cell death, especially apoptosis, was observed early in the study of NTC [12, 98, 115]. Apoptosis, which is prominent during development, is propogated through signaling cascades that eventually converge on and activate cysteine proteinases, the caspases, which ultimately cause cell death through cleavage of their substrate proteins [116, 117]. At the boundary of the rhombencephalon and mesencephalon, extensive apoptosis is observed—both in the non-neural surface ectoderm and the neuroepithelium—before the neural folds are apposed and fused (Fig. 4). Because this pattern coincides with CNC generation, it has long been assumed that apoptosis contributes to CNC development, although its role has not been clearly determined [118]. Apoptosis is also extensive at the anterior neural ridge (ANR), which is the boundary region of the most rostral prosencephalic region, and CNCs do not originate in this region. The role of apoptosis in the ANR is not yet known.

Mice lacking intrinsic apoptotic pathway genes (*apaf-1*,* caspase-3*, or* caspase-9*) or harboring a mutant form of *cytochrome*-*c* that cannot activate apoptotic pathway but is intact for electron transport, or double-knockout mice for JNK1/JNK2 genes, exhibit cranial NTDs, including exencephaly [119–123]. These results indicate that regulation of apoptosis is involved in successful cranial NTC. Although many of the boundary cells responsible for fusion undergo apoptosis, inhibiting apoptosis does not affect the fusion process itself [100, 118]. Recently, live-imaging analysis revealed that in the absence of apoptosis mediated by caspase activation, DLHP formation and the flipping of the neural ridge are perturbed in MHNP, thus delaying cranial NTC [100]. This suggests that apoptosis by caspase activation promotes the smooth progression of neural plate morphogenesis during cranial NTC.

It is not yet clear how apoptosis mediated by caspase activation (which occurs mainly in the boundary regions) achieves this, nor whether apoptosis acts permissively or instructively on the progression of NTC. Apoptosis mediated by caspases may instructively help to generate forces that promote epithelial sheet morphogenesis, as shown in other model organisms and in cell culture systems [124–126]. To determine this conclusively in mice would require new tools to inhibit or induce caspase activation and apoptosis, with precise control over region and timing. It is also worth investigating whether apoptosis and caspase activation in the boundary regions acts through the adjacent neuroepithelium and surface ectoderm by releasing signaling molecules such as growth factors, small-molecule hormones, and fatty acids [127–132].

Apoptosis occurs continuously from the beginning through to the final step of NTC, including the entire remodeling process in which the neuroectoderm and the outer non-neural ectoderm are separated and arranged to make a rigid neural tube. However, the contribution of apoptosis to this remodeling process is still unclear, as it is in other tissue-fusion processes in which extensive apoptosis is observed [101, 133]. In cultured mouse embryos, it was reported that chemically inhibiting apoptosis does not affect the fusion or the separation of the neuroectoderm and epidermis [118], suggesting that apoptosis is dispensable for fusion and separation.

#### Non-apoptotic cell death and autophagy

Nevertheless, it is not yet clear whether cell death itself is non-essential in the remodeling process, because even in apoptosis-deficient embryos, alternate forms of cell death (non-apoptotic cell death) can occur [134, 135]. To clarify the impact of programmed cell death itself on NTC, it is necessary to determine whether non-apoptotic cell death such as caspase-independent cell death or autophagic cell death occurs in these embryos during NTC, and what actually causes the cells to die in the process.

Cell death is often accompanied by autophagy [136]. The role of autophagy during the cranial NTC remains to be elucidated. Mice deficient for *ambra1*, which is necessary for beclin1-dependent autophagosomal formation during murine development, exhibit exencephaly and spina bifida [137]. Although macroautophagy is mainly mediated by Atg5 or Atg7, mutant mice deficient for those genes do not show any apparent developmental defects in NTC [138, 139]. Thus, NTC does not require Atg5/Atg7-dependent autophagy but does require the recently identified beclin1-mediated alternative autophagic pathway [140]. Interestingly, *ambra1* KO embryos showed increased Shh signaling in the neural tube, suggesting that there might be an interaction between the regulation of Shh signaling and the Ambra 1 protein, and that this may be the cause of NTDs in these embryos [137, 141]. Further studies will be needed to reveal the complex interplays between cell death, autophagy, and cell differentiation programs during cranial NTC.

### Remodeling and the integrity of the neural tube and epidermis

Little is known about cellular and molecular mechanisms of remodeling in the midline after fusion (Fig. 1f) [101]. During remodeling, dynamic cellular behaviors such as cell rearrangement, cell mixing, cell proliferation, and cell death are expected and indeed observed as mentioned above—but not precisely understood. Thus, it is difficult at present to assess the significance and normal characteristics of this remodeling. Detailed studies of these cellular behaviors are needed to determine the precise remodeling mechanisms that, if disrupted, would cause the neural tube to reopen, an event that results in exencephaly or spina bifida.

In fact, reopening of the neural tube might be considered a remodeling failure. The tumor suppressor gene *neurofibromatosis type 2* (*Nf2*) likely contributes to the remodeling steps that prevent the neural tube from reopening: the *Nf2* gene product regulates the assembly of apico-lateral junctional complexes in the neuroepithelium [142]. Eliminating *Nf2* specifically in developing neuroepithelium from E8.5 does not affect the initial fusion process, but the cranial neural tube reopens after E9.5, causing exencephaly. Thus, establishing the proper cell–cell adhesion structures during remodeling seems to be important for keeping the neural tube closed, although this concept has not been directly tested by experimental manipulation of the remodeling.

### Genetic background affects susceptibility

The penetrance of exencephalic phenotypes in the presence of genetic or environmental perturbation can vary according to mouse genetic background [9, 143, 144]. For instance, many knockout NTD mice maintained on the 129-dominant background exhibit exencephaly; while those on a C57B6L background do not—although the opposite has been reported in other cases (see [9]). Mice that differ in NTD phenotype according to their genetic background include mutants or knockouts for transcription factors such as *p53, Cart1, Sall2*, and *splotch* (*Sp* ^*2H*^) (pax3) [17, 40, 78, 145], apoptosis regulators such as *caspase*-*3* and *apaf*-*1* [146–148], growth factor signaling (*Noggin*) [25, 26], cellular trafficking [149, 150], and chromatin modifiers (*Jumonji*) [151, 152]. Presumably, multifactorial causes underlie the phenotypic differences and penetrance in these cases.

Interestingly, the mode of closure 2 appears to affect susceptibility to exencephaly under certain kinds of genetic or environmental perturbation in cranial NTC [15, 17]. Although the point where closure 2 begins is usually at the forebrain-midbrain boundary (FMB) (Fig. 2b, c), this location varies among mouse strains [10, 15–17]. The SELH/Bc strain, for example, likely lacks closure 2 [15]; the forebrain is sealed only by closure 1 and closure 3, and this strain has a spontaneous exencephaly rate of about 20 %. This is not the only example. Closure 2 begins caudal to the FMB in the DBA/2 strain, and rostral to the FMB in the NZW strain [17]. Interestingly, a *Sp* ^*2H*^ (*pax3*) mutation introduced into DBA/2 background is less susceptible to exencephaly, but increases the susceptibility in NZW background [17]. These data have prompted the suggestion that, as the starting site of closure 2 becomes more rostral, susceptibility to exencephaly increases in the presence of genetic perturbation or teratogenic agents [14, 17, 153]. It is not yet clear what causes the differences in closure 2 position, or how a more rostral site increases the risk of exencephaly.

### A kinetic view of cranial NTC: Is there a closure deadline?

**Fig. 5:**
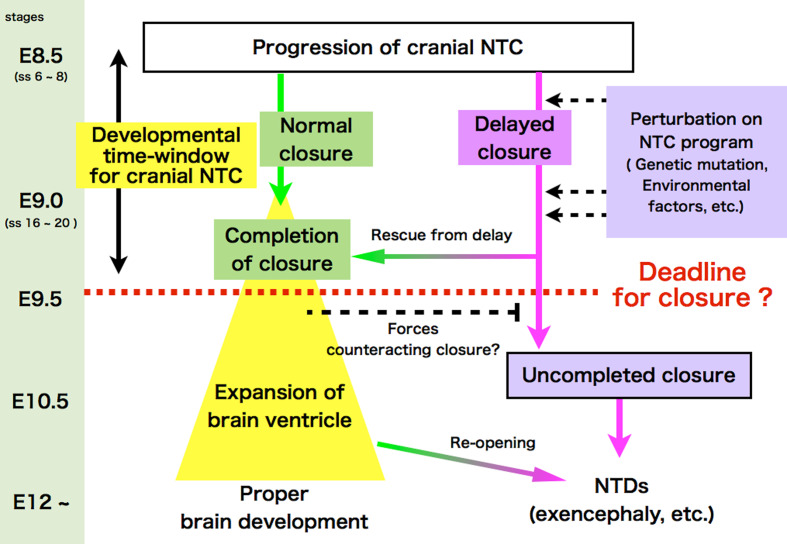
Developmental time-window model for cranial NTC. NTC must be completed by a hypothetical developmental deadline (about somite stage 20), when forces incompatible with NTC may arise. Any perturbation on NTC program could delay NTC. Even when closure is delayed, the embryo can develop without NTDs, as long as NTC can be completed before the deadline (shown as “rescue form delay”). However, if closure is not completed by the deadline, cranial NTC ends in failure to close at the MHNP, resulting in cranial NTDs such as exencephaly

The more rostrally positioned closure 2 results in increase of the length of the MHNP that must be sealed by closures 1 and 2. This presumably lengthens the time it takes to close the MHNP, thus delaying the completion of NTC. We performed a live-imaging observation of delayed closure and perturbed neural fold movement in the absence of apoptosis. On the basis of the results, we proposed a working model of a deadline for cranial NTC. This “developmental time window” hypothesis holds that forces counteracting closure are generated and eventually surpass those promoting closure as embryonic brain development proceeds. In normal development, cranial NTC completes before counteracting forces become strong enough to interfere with closure (Fig. 5) [100]. However, if the progress of closure is delayed, whether due to genetic, environmental, or physical disturbances, cranial NTC fails—or the closure reopens as observed in a live-imaging analysis [100]—due to the stronger counteracting forces. This model explains why a disturbance in the NTC process would more severely impact mice with a rostrally positioned closure 2 or with delayed closure. The penetrance of NTDs varies in mice harboring mutations known to cause them. Viewing NTC kinetically from this model may explain the variable phenotypic penetrance.

### Live imaging of cranial NTC with functional reporters for cellular or signaling activities

Causal relationships between genetic mutations and NTDs have been identified by developing hundreds of mutant mouse models [4, 9]. In many cases, the linkage between a genetic mutation and the consequent NTD has not been clarified, since the complexity and dynamics of cranial NTC cannot be captured solely by static methods. Recently developed live-imaging analysis allows for more precise investigations. Using this approach, researchers have revealed how PCP signaling, cytoskeletal dynamics, or cellular behavior acts on neural tube morphogenesis in various animal models, including ascidian, fish, amphibians, and birds, whose embryos are more accessible for such analyses than are mammalian embryos [36, 43, 74, 154–157]. Such analyses will also help to understand basic mechanisms of the mammalian cranial NTC, which is much more complex than non-mammalian vertebrates [11].

Furthermore, live-imaging analysis with functional reporters for biological signals or molecules can now allow us to visualize and monitor the activities or behaviors of signaling molecules within living cells. Although it has long been difficult to generate transgenic mice that stably expresses genetically encoded fluorescent reporters monitoring biological signaling activities, several groups have succeeded to generate them [100, 158–160]. With new fast-scanning confocal microscopy methods for high-resolution observation of living embryos, it has now become possible to observe real-time cell signaling during mammalian cranial NTC [99, 100]. This has revealed unexpected, differential modes of apoptosis occurring during NTC [100]. It would be interesting to visualize neural plate morphogenesis and the specific signaling pathways responsible for closure. Further development of these new methods of analysis will allow us to gain new insights into the mechanics and dynamics of cranial NTC and etiology of NTDs.

### Nutrition, metabolism, and epigenetic regulation

We have summarized factors contributing to the process of cranial NTC, focusing on neural plate morphogenesis and cell–cell/tissue–tissue interactions. In addition to these embryonic mechanisms of NTC, we want to conclude by mentioning two other aspects that are important when considering the etiology of human NTDs; contribution of maternal nutritional factors including folic acid, and epigenetic regulation [2, 7, 161].

Among maternal nutrient factors, the preventive effects of folic acid on human NTD risk have been well established, and several countries mandate folic acid fortification of the grain supply [5, 6]. However, how folic acid contributes to normal NTC or prevents NTDs is not well known. There are so far six genes that are identified to be responsible for folate transport in mammals; the glycosyl-phosphatidylinositol-anchored folate receptors (*Folr 1, Folr 2, Folr 3, and Folr 4*) [162, 163]*, the bidirectional reduced folate carrier 1* (*RFC1; also known as SLC19A1*) [164], and *protom coupled folate transporter* (*PCFT*) [165]. Mice deficient for either *Folr1* or *RFC* exhibit severe growth retardation and embryonic lethality before the beginning of NTC [162, 166]. Supplementation with a high amount of folates to pregnant mothers allows those mutant embryos to survive to birth, suggesting that folate transport from amniotic fluid to embryos is essential for embryonic growth [166, 167]. Embryos from mothers supplemented with modest levels of folates are rescued from early embryonic lethality but still exhibit NTDs [168]. Interestingly, *Folr1* is strongly expressed in the dorsal tips of neural folds during NTC, implying that developmental events in those regions, including neural crest generation, may require a high amount of available folates [169, 170]. These lines of evidence together suggest that the adequate amount of folates available for embryos is a crucial factor throughout developmental stages from gastrulation to neurulation. However, it is not yet clear how this is related to preventive effects of folates on NTDs. There are both folate-sensitive and folate-resistant NTDs mouse models. Furthermore, recent studies suggest that excessive folic acid intake is deleterious to several NTD mouse mutants, and even to normal embryogenesis [171, 172]. These lead to general concerns about unintended consequences of folic acid supplementation [5, 6].

Folate availability affects the one-carbon metabolism that supplies, as its name suggests, the one-carbon groups required for de novo synthesis of purines and thymidylate, or methylation [7]. Indeed, availability of folate impacts both nucleotide synthesis and DNA methylation [173–175]. Interestingly, deficient DNA methylation leads to cranial NTDs [176, 177]. Such epigenetic regulation by methylation may also be involved in the higher rate of exencephaly seen in female embryos [177, 178]. A recent study reported that loss-of-function mutations in the glycine-cleavage system, which is an important component of folate one-carbon metabolism in mitochondria, predispose to NTDs in humans and in mice [179]. This suggests that functional folate one-carbon metabolism itself is crucial for NTC. How folate metabolism and epigenetic regulation fit into the developmental NTC mechanism remains to be determined in future studies.

## Conclusions and perspective

Cranial NTC is a fascinating, dynamic process that is crucial to the development of functional brain. In this review, we have attempted to clarify the mechanisms of cranial NTC. Normal developmental programs required for cranial NTC include neural plate patterning, signaling systems responsible for tissue movement or fusion, and mechanisms responsible for the coordination of cell division, cell differentiation, and cell death. By examining these developmental programs, it will be possible to understand the mechanical and kinetic aspects of closure that may largely affect the occurrence of cranial NTDs. Newly emerging techniques including functional live-imaging analysis now allow researchers to analyze the interactions between signaling activity and morphological changes in detail in various model organisms including mice. With these tools, it may be possible to determine precisely when and how mutations disrupt normal developmental programs and produce NTDs. This knowledge may also help us to understand the action of teratogenic drugs and to find ways to prevent NTDs [144].

## Abbreviations

NTC: Neural tube closure
NTDs: Neural tube defects
MHP: Medial hinge point
DLHP: Dorsolateral hingepoint
ss: Somite stage
ANP: Anterior neuropore
MHNP: Midbrain-hindbrain neuropore
BMPs: Bone morphogenetic proteins
SHH: Sonic Hedgehog
PCP: Planar cell polarity signaling
AJs: Adherens junctions
Grhl: Grainyhead-like
Par1: Protease-activated receptor 1
CNC: Cranial neural crest cell
FMB: Forebrain-midbrain boundary
ANR: Anterior neural ridge
Apaf-1: Apoptotic protease activating factor 1
JNK: c-Jun N-terminal kinase
Ambra 1: Activating molecule in beclin-1-regulated autophagy
